# Construction of Prognostic Risk Prediction Model of Oral Squamous Cell Carcinoma Based on Nine Survival-Associated Metabolic Genes

**DOI:** 10.3389/fphys.2021.609770

**Published:** 2021-03-16

**Authors:** Zhen-Dong Huang, Yang-Yang Yao, Ting-Yu Chen, Yi-Fan Zhao, Chao Zhang, Yu-Ming Niu

**Affiliations:** ^1^Center for Evidence-Based Medicine and Clinical Research, Taihe Hospital, Hubei University of Medicine, Shiyan, China; ^2^Department of Stomatology, Southern Medical University, Guangzhou, China; ^3^The First Affiliated Hospital of Xinjiang Medical University, Ürümqi, China; ^4^Department of Oral and Maxillofacial Surgery, Taihe Hospital, Hubei University of Medicine, Shiyan, China

**Keywords:** oral squamous cell carcinoma, metabolic gene, prognostic model, risk score, prediction model

## Abstract

The aim was to investigate the independent prognostic factors and construct a prognostic risk prediction model to facilitate the formulation of oral squamous cell carcinoma (OSCC) clinical treatment plan. We constructed a prognostic model using univariate COX, Lasso, and multivariate COX regression analysis and conducted statistical analysis. In this study, 195 randomly obtained sample sets were defined as training set, while 390 samples constituted validation set for testing. A prognostic model was constructed using regression analysis based on nine survival-associated metabolic genes, among which PIP5K1B, NAGK, and HADHB significantly down-regulated, while MINPP1, PYGL, AGPAT4, ENTPD1, CA12, and CA9 significantly up-regulated. Statistical analysis used to evaluate the prognostic model showed a significant different between the high and low risk groups and a poor prognosis in the high risk group (*P* < 0.05) based on the training set. To further clarify, validation sets showed a significant difference between the high-risk group with a worse prognosis and the low-risk group (*P* < 0.05). Independent prognostic analysis based on the training set and validation set indicated that the risk score was superior as an independent prognostic factor compared to other clinical characteristics. We conducted Gene Set Enrichment Analysis (GSEA) among high-risk and low-risk patients to identify metabolism-related biological pathways. Finally, nomogram incorporating some clinical characteristics and risk score was constructed to predict 1-, 2-, and 3-year survival rates (C-index = 0.7). The proposed nine metabolic gene prognostic model may contribute to a more accurate and individualized prediction for the prognosis of newly diagnosed OSCC patients, and provide advice for clinical treatment and follow-up observations.

## Background

Among head and neck squamous carcinoma worldwide, > 90% patients suffered from oral squamous cell carcinoma (OSCC) (Marur and Forastiere, 2016; Henley et al., 2020), which was a life-threatening disease with high morbidity and mortality. There were an estimated over 350,000 new diagnoses and more than 175,000 deaths worldwide in 2018 (Ferlay et al., 2019). OSCC was the most common type of oral malignancy, with half a million new cases diagnosed each year in India (Gupta et al., 2016). It was widely demonstrated that the development of OSCC was strictly influenced by unhealthy habits such as alcohol abuse, tobacco, tobacco-derivate, chewing betel nut (Ng et al., 2017; Falzone et al., 2019), and human papillomavirus (HPV) infection (Chi et al., 2015). OSCC was generally asymptomatic in the early stages, which can lead to late diagnosis, extensive lesions, and potential metastases, while lymph node metastases were widely recognized as a major cause of poor survival (Gharat et al., 2016; Bray et al., 2018; Velmurugan et al., 2020). The 5-year survival for OSCC has been reported approximately 50% (Warnakulasuriya, 2009; Taghavi and Yazdi, 2015; Stanciu et al., 2020). Despite intervention with advanced treatment regimens like chemoradiotherapy, surgery, EGFR (epidermal growth factor receptor) inhibitors and COX-2 inhibitors, and photodynamic therapy, survival rate of OSCC has not improved significantly in recent decades (Rhodus et al., 2014; Chi et al., 2015; Gupta et al., 2016). In addition to drug resistance, complications resulting from the death of non-characteristic cells as a result of these treatments also contribute to the low survival rate. Therefore, in order to better adjust the treatment intensity and avoid serious complications caused by overtreatment, it was urgent to study potential prognostic markers. Relevant study showed that metabolic phenotypes provide information on patient prognosis and the treatment of cancer (Vander Heiden and DeBerardinis, 2017). As we know, normal cell metabolism was based on normal signaling pathways and basic metabolites, and many studies have shown that cell metabolism changes in cancer. The abnormal activity of these pathways, as one of the most significant events in cancer, accelerated the development of tumor and arouses great interest in tumor metabolism (Vander Heiden and DeBerardinis, 2017; Fakhri et al., 2020). Therefore, metabolic genes had been widely concerned by cancer researchers, and related studies such as hepatocellular carcinoma (Yang et al., 2020), gliomas (Qi et al., 2019), prostate cancer (Carbonetti et al., 2019), and soft tissue sarcoma (Gu et al., 2020) have been reported. The specific mechanism between cancer and metabolic reprogramming has been widely studied, but as far as OSCC was concerned, the specific mechanism is not well understood.

Current diagnostic gold standard of OSCC was biopsy, and therapeutic schedule and prognostic predictions usually according to the TNM stage. However, TNM staging could not meet the needs for the selection of treatment options and prognosis prediction. In this study, a metabolic gene prognostic model was constructed based on regression analysis to improve prognosis and guide the meticulous design of better treatments. Regression analysis including univariate COX regression analysis, least absolute shrinkage and selection operator (Lasso) regression analysis and multivariate COX regression analysis were widely used in the study of cancer prognosis such as hepatocellular carcinoma (Mule et al., 2020), lung adenocarcinoma (Zhu et al., 2020), bladder cancer (Jiang et al., 2020), and ovarian cancer (Ding et al., 2020). However, research into OSCC should be supplemented. The data downloaded from The Cancer Genome Atlas (TCGA), which is the ambitious and successful cancer genomics programs with over 30 different types of cancer, 11,000 specimens and related information (available genomic sequence, expression, methylation, and copy number) (Cooper et al., 2018). Regression analysis were used to determine the gene signatures and construct the prognostic model. Statistical analysis was conducted to evaluate the prognostic value. This study may be beneficial to the prediction of clinical prognosis and assist in the formulation of OSCC treatment plans.

## Materials and Methods

### Data Collection and Preprocessing

Gene expression profile and clinical data from oral cavity (alveolar ridge, buccal mucosa, floor of mouth, tongue, lip, oral cavity, and hard palate) were selected, while samples from larynx, bones, joints, articular cartilage of other and unspecific sites were excluded. Furthermore, metabolism-associated gene sets with Kyoto Encyclopedia of Genes and Genomes (KEGG) (Kanehisa et al., 2016) pathways in the background were obtained at the Gene Set Enrichment Analysis (GSEA) website^1^. After pretreatment, a matrix with samples and metabolism-related gene expression values was used for subsequent analysis.

### Identification of Primary Differentially Expressed Genes

This part was carried out in the “limma” package, a popular choice for gene discovery, contained particularly strong facilities for reading, normalizing, and exploring such data (Ritchie et al., 2015). The “limma” perform differential expression analyses of RNA sequencing (RNA-seq) data based on a linear model implemented, | log_2_ fold change (FC)| and false discovery rate (FDR) were generally used to draw a conclusion (Ritchie et al., 2015). Log-fold-changes represented the transcriptional signature and FDR-values assessed the significance of the observed expression changes. The genes with | log_2_ FC| > 0.5 and FDR < 0.05 were thought to be primary differentially expressed genes (DEGs) in this study. This result was visualized as a volcano plot.

### Prognostic Model Construction and Evaluation Based on Identified Survival-Related DEGs

All OSCC samples were divided into training set and validation set. Training set randomly obtained was used to construct the prognosis model, while validation set to test. There were three main steps used to identify survival-related DEGs and constructed prognostic model: univariate COX regression analysis, Lasso regression analysis and multivariate COX regression analysis. The Lasso regression analysis, a machine learning algorithm with the property that it simultaneously performs variable selection and shrinkage (Tibshirani, 1997; Goeman, 2010), was widely used to construct a prognostic model (Tang and Yin, 2020). The procedure above was conducted in “glmnet” and “survival” packages of R (Friedman et al., 2010). The gene signatures obtained by stepwise screening of metabolism-related DEGs through COX regression analysis and Lasso regression analysis were considered as survival-related DEGs, key genes. In other words, DEGs with *P* < 0.05 were considered as key genes in both COX regression analysis and Lasso regression analysis. In order to construct the prognosis model, key gene and its correlation coefficient need to be determined based on multivariate COX regression analysis: risk score = $\sum_{i=1}^{n}\beta_{i}\times \text{exp}\,\left(G_{i}\right)$, where n is the number of genes identified for the multivariate COX regression model; exp(G_*i*_) is the expression value of gene i; and β*i* refers to the coefficient for gene i (risk score = $\sum_{i=1}^{n}c\,o\,e\,f\,i\times x_{i}$ In which the coefi is the coefficient, and *x*_*i*_ is the expression value of each selected gene). So, each patient had a parameter: risk score. Patients in the training group were divided into high and low risk groups according to the median risk score. Patients in the validation set were also divided into two groups based on the median risk score of the training set. Then, all patients labeled “high-risk” or “low-risk.” The prognostic model was evaluated based on overall survival (OS). Moreover, ROC curve was applied to confirm prognostic efficiency in “survivalROC” package of R package. The prognostic model was also evaluated synchronously based on validation set.

### Kaplan–Meier Survival Analysis

Kaplan–Meier survival analysis, a non-parametric method, was used to determine the relationship between the expression profile of one or more genomes and survival time. In the section, survival curves based on OS were plot based on two groups (“high-risk” vs. “low-risk”), while log-rank test can draw a conclusion that if there was statistical significance between groups (*P* < 0.05). To assess the relationship between key genes and OSCC patients, we conducted the Kaplan–Meier survival analysis and log-rank test using the “survival” package of R software in training set and validation set.

### Independent Prognostic Analysis

In order to explore the impact of clinical factors (such as age, gender, grade, stage, T, N, and M) on the prognostic model, independent prognostic analyses were conducted based on OS. In addition, the effect of risk score as an influencing factor on prognosis was also evaluated based on regression analysis. Missing clinical feature data over 50% of the total sample will not be included. The clinical feature with *P*-value < 0.05 was considered significant, and was considered to be an independent prognostic factor. Both training and validation sets were used for independent prognostic analyses based on univariate and multivariate COX regression analyses that were conducted using the “survival” package.

### The Prognosis Analysis of Patients Received Different Treatments

The clinical outcome of OSCC patients depends not only on the individual characteristics of each patient, but also on the effectiveness of treatment. OSCC patients received radiotherapy and pharmacotherapy were subjected to prognostic analysis, which included Kaplan–Meier survival analysis, and the risk scores of patients received different treatments were compared. The “ggplot2” and “survival” package of R software were used.

### Immune Infiltration Analysis

Increasing evidence has elucidated that tumor-infiltrating immune cells not only play a crucial role in tumor progression and therapeutic efficacy but also show clinical significance in predicting outcomes. Therefore, it was necessary to investigate the correlation between risk score and tumor infiltrating immune cells (B cells, CD4^+^ T cells, CD8^+^ T cells, neutrophils, macrophages, and dendritic cells) based on Tumor Immune Estimation Resource (TIMER^2^) database, which a website that allowed users to interactively explore the associations between immune infiltrates and a wide spectrum of factors such as gene expression, somatic mutations, clinical outcomes and somatic copy number alterations (Li et al., 2016, 2017). If the *P*-value was less than 0.05, we would expect a significant correlation between the risk score and the infiltration of immune cells. To further explore the differences in immune cell infiltration between the high and low risk groups, the immune scores of the two groups were calculated. When *P*-value was less than 0.05, the difference between the high and low risk groups was considered statistically significant. To verify the robustness of the test set results, the validation set was also subjected to immune infiltration analysis.

### Construction of the Nomogram and Internal Validation

The construction of nomogram based on the OS of OSCC patients. Clinical factors such as age, gender, grade, stage, T, N, and M were considered. However, not all clinical features were available, and more than half of the missing data will be excluded. The model was internally validated by bootstrap resampling with 1,000 replicates to evaluate reliability and stability and C-index draw a conclusion. The concordance index (C-index) can measure the capacity of the model to discriminate patients with different outcomes: the higher the C-index, the more significant the model was about survival outcome (Huitzil-Melendez et al., 2010). Furthermore, calibration curves were plotted. *P*-value threshold < 0.05 was set as statistically significant.

### Gene Set Enrichment Analysis

In order to understand the expression pathways of these key genes, these key genes were defined as a set of genes for GSEA, which is a computational tool commonly employed to interpret gene expression data by evaluating whether a pre-defined set of genes demonstrates statistically significant (Subramanian et al., 2005; Li et al., 2020). The terms with *P*-value < 0.05 obtained in GSEA was considered significant and the top 10 terms were visualized using “ggplot2” package.

## Results

### Data Preprocessing and Identification of Primary DEGs

Raw microarray data including 390 OSCC and 32 normal oral tissues download from TCGA. After preprocessing, a matrix consisting of 1,723 metabolic gene signatures based on KEGG and their expression values in 422 samples was obtained. 452 metabolism-related genes with | log_2_ FC| > 0.5 and FDR < 0.05 were thought to be primary DEGs, including 209 down-regulated genes and 243 up-regulated genes. Heatmap of DEGs was displayed in Supplementary Figure 1 and the volcano plot in Supplementary Figure 2.

### Prognostic Model Construction and Evaluation Based on Identified Survival-Related DEGs

**FIGURE 1:**
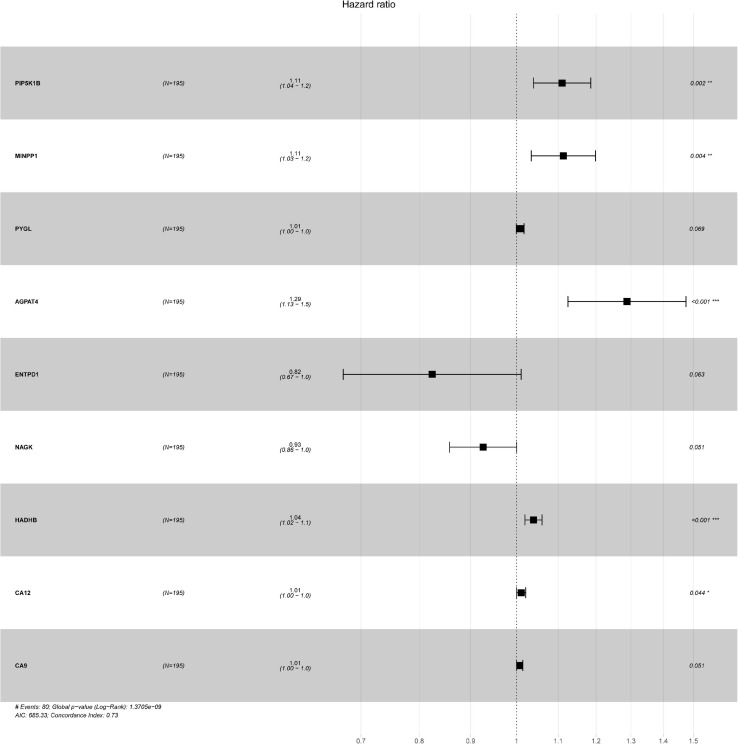
The forest plots of hazard ratio for each key genes, including PIP5K1B, MINPP1, PYGL, AGPAT4, ENTPD1, NAGK, HADHB, CA12, and CA9. ^∗^*P* < 0.05 was considered statistically significant, and concordance Index = 0.73.

Oral squamous cell carcinoma samples were randomly divided into training set and validation set. In this study, 195 randomly obtained sample sets were defined as training sets, while 390 samples constituted validation set for testing sets. A total of 58 DEGs were selected *via* univariate Cox regression analysis with the threshold was set to *P* < 0.05. To further identify the 58 DEGs that were significantly correlated with the prognosis of OSCC patients, Lasso regression with 10-fold cross-validation was performed, then 13 genes from 58 DEGs were obtained. Supplementary Figure 3A illustrated Lasso coefficients profiles and Supplementary Figure 3B illustrated Lasso regression with 10-fold cross-validation obtained 13 prognostic genes using minimum lambda value. Finally, multivariate COX regression analysis identified nine survival-related DEGs as key genes [PIP5K1B (coef = 0.104533195), MINPP1 (coef = 0.107435564), PYGL (coef = 0.008286949), AGPAT4 (coef = 0.253141308), ENTPD1 (coef = −0.193545659), NAGK (coef = −0.076520344), HADHB (coef = 0.038865293), CA12 (coef = 0.010700128), and CA9 (coef = 0.007224148)] for constructing prognostic model in Table 1. PIP5K1B, NAGK, and HADHB significantly down-regulated, while MINPP1, PYGL, AGPAT4, ENTPD1, CA12, and CA9 significantly up-regulated in Table 2. Additionally, the forest pot was illustrated in Figure 1. PIP5K1B, MINPP1, AGPAT4, HADHB, and CA12 has been shown to be significantly associated with survival (^∗^*P* < 0.05 was considered statistically significant). The risk score for each OSCC sample was calculated based on the key genes prognostic signature using the following formula: risk score = 0.104533195 × exp(PIP5K1B) + 0.107435564 × exp(MINPP1) + 0.008286949 × exp(PYGL) + 0.253141308 × exp(AGPAT4) + (-0.193545659) × exp(ENTPD1) + (−0.076520344) × exp(NAGK) + 0.038865293 × exp(HADHB) + 0.010700128 × exp(CA12) + 0.007224148 × exp(CA9) in Table 1. Patients with their own risk scores in the training set were then marked as high or low risk based on median risk score. It must be emphasized that the grouping parameters of the validation set were also the median risk score of the patients in the training set.

**TABLE 1:** Multivariate COX regression analysis of key genes.

Gene symbol	coef	HR	HR.95L	HR.95H	*P*-value
PIP5K1B	0.104533195	1.110192246	1.039647305	1.185523992	0.001804018
MINPP1	0.107435564	1.113419114	1.034382729	1.198494609	0.004238951
PYGL	0.008286949	1.008321381	0.999367747	1.017355233	0.068608706
AGPAT4	0.253141308	1.288065277	1.125134916	1.474589523	0.000243795
ENTPD1	−0.193545659	0.824032211	0.671932139	1.010561997	0.06302064
NAGK	−0.076520344	0.926334069	0.857815932	1.0003251	0.050976973
HADHB	0.038865293	1.039630428	1.01945738	1.060202661	0.000101285
CA12	0.010700128	1.010757579	1.000267542	1.021357628	0.044407902
CA9	0.007224148	1.007250305	0.99998368	1.014569734	0.050518646

*coef, coefficient; HR, hazard ratio.*

**TABLE 2:** The magnitude and significance of nine key genes.

Gene	Log FC	*P*-value	Up/Down
PIP5K1B	−1.549576334	0.00040578	Down
MINPP1	1.059847424	3.05E-12	Up
PYGL	1.365791775	1.67E-09	Up
AGPAT4	1.028209169	3.95E-07	Up
ENTPD1	0.557752228	4.92E-05	Up
NAGK	−0.546036237	0.000224183	Down
HADHB	−0.781443169	1.44E-05	Down
CA12	0.621064462	0.008090628	Up
CA9	6.363642335	1.63E-18	Up

**FIGURE 2:**
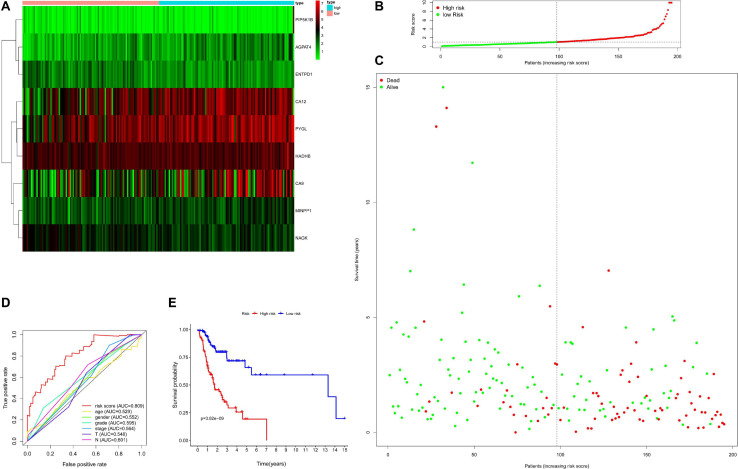
Evaluation for prognostic model based on training set including 195 samples. Panel **(A)** indicates heatmap showing the nine key genes expression distribution in training cohorts, each column represented one patient, panel **(B)** indicates risk score distribution, each point represented one patient sorted by the rank of the risk score, red and green represent high-risk and low-risk patients, respectively, panel **(C)** indicates scatter diagram of survival status the sample, red and green represent dead and alive, respectively, panel **(D)** indicates efficiency assessment of age, gender, grade, stage, T, N, and risk score based on ROC curve, and panel **(E)** indicates Kaplan–Meier survival curves estimating OS.

**FIGURE 3:**
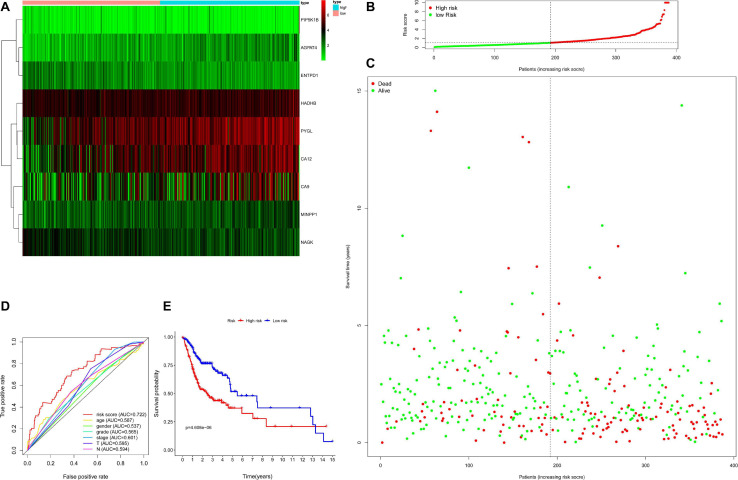
Evaluation for prognostic model based on validation set including 390 samples. Panel **(A)** indicates heatmap showing the nine key genes expression distribution in testing cohorts, each column represented one patient, the green to red spectrum indicates low to high gene expression, panel **(B)** indicates risk score distribution, each point represented one patient sorted by the rank of the risk score, red and green represent high-risk and low-risk patients, respectively, panel **(C)** indicates scatter diagram of survival status the sample, red and green represent dead and alive, respectively, panel **(D)** indicates efficiency assessment of age, gender, grade, stage, T, N, and risk score based on ROC curve, and panel **(E)** indicates Kaplan–Meier survival curves estimating OS.

Then, the accuracy and sensitivity of prognostic model were evaluated: Figure 2A illustrated the nine key genes expression distribution in training cohorts, each column represented one patient, Figure 2B illustrated risk score distribution, each point represented one patient sorted by the rank of the risk score, red and green represent high-risk and low-risk patients, respectively, Figure 2C indicates scatter diagram of survival status the sample, red and green represent dead and alive, respectively. Optimal cut-off values were used to classify the subgroups of patients with high and low risk scores. The dotted line represents the cut-off point for the median risk score, which is used to classify patients into low-risk and high-risk groups. Prognostic predicted efficiency of risk score (AUC = 0.809) was better than other clinical factors including age (AUC = 0.529), gender (AUC = 0.552), grade (AUC = 0.595), stage (AUC = 0.564), T (AUC = 0.548), and N (AUC = 0.601) based on ROC curves (more than half of the M clinical data was missing and therefore not included in the study) in Figure 2D. The Log-rank testing of the Kaplan–Meier curve were applied to figure out the difference of OS rate between the high-risk and low-risk groups that lower OS in high-risk group (*P* < 0.05) in Figure 2E. In order to verify the value of the prognostic model, the validation set was also used to evaluate the prognostic model. Figures 3A–C illustrated the nine gene expression distribution, risk distribution and survival status based on the validation set, respectively: the significant differences between the high and low risk groups, and the number of patients dying increased as the risk score increased. The AUC of the clinical features included in the study (age, gender, grade, stage, T, and N) was lower than the risk score (AUC = 0.722), indicating that the risk score had more accurate predictive power in Figure 3D. The survival analysis based on the validation set (Figure 3E) showed that the survival probability of both groups decreased with the increase of time, but the survival probability of the high-risk group decreased more significantly and the survival probability of the two groups was significantly different (*P* < 0.05). The prognostic model performed well in both the training set and the validation set.

### Independent Prognostic Analysis

**FIGURE 4:**
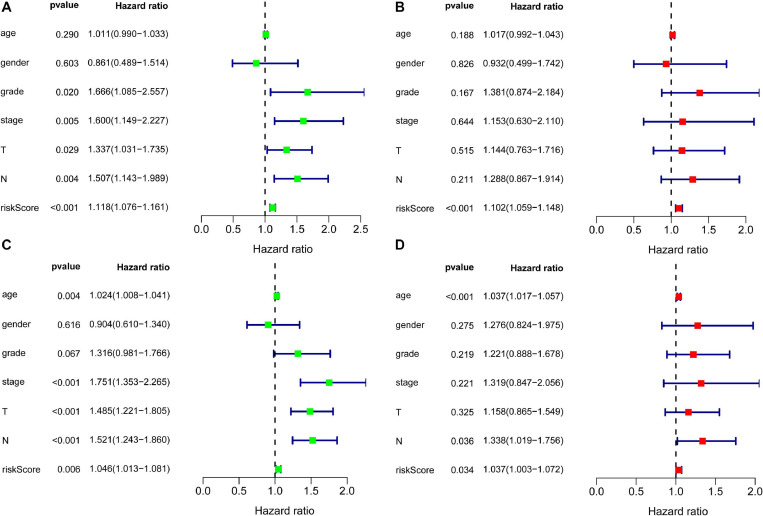
Forest plots for identification of the prognosis-related clinical factors. Panel **(A)** indicates univariate COX regression analysis based on training set, panel **(B)** indicates multivariate COX regression analysis based on training set, panel **(C)** indicates univariate COX regression analysis based on validation set, and panel **(D)** indicates multivariate COX regression analysis based on validation set.

Since the missing clinical data of M was more than 50%, the clinical factors included in the independent prognostic analysis included age, gender, grade, stage, T, and N. Independent factors were sought through COX regression analysis of univariate and multivariate. COX regression of univariate analysis based on training set revealed that grade (hazard ratio (HR) 1.666, 95% confidence interval (CI) 1.085–2.557; *P* = 0.020), stage (HR: 1.600, 95% CI: 1.149–2.227, *P* = 0.005), T (HR: 1.337, 95% CI: 1.031–1.735, *P* = 0.029), N (HR: 1.507, 95% CI: 1.143–1.989, *P* = 0.004), and risk score (HR: 1.118, 95% CI: 1.076–1.161, *P* < 0.001) were considered independent prognostic factors (*P* < 0.05) (Figure 4A). COX regression of multivariate analysis based on training set demonstrated risk score (HR: 1.102, 95% CI: 1.059–1.148, *P* < 0.001) was an independent prognostic factor (Figure 4B).

To further validate the risk score, independent prognostic analyses were also performed in the validation set. The result of independent prognostic factors (*P* < 0.05), including age (HR: 1.024, 95% CI: 1.008–1.041, *P* = 0.004), stage (HR: 1.751, 95% CI: 1.353–2.265, *P* < 0.001), T (HR: 1.485, 95% CI: 1.221–1.805, *P* < 0.001), N (HR: 1.521, 95% CI: 1.243–1.860, *P* < 0.001), and risk score (HR: 1.046, 95% CI: 1.013–1.081, *P* = 0.006), were demonstrated using univariate COX regression analysis in Figure 4C. The result of multivariate COX regression analysis illustrated in Figure 4D: age (HR: 1.037, 95% CI: 1.017–1.057, *P* < 0.001), N (HR: 1.338, 95% CI: 1.019–1.756, *P* = 0.036), and risk scores (HR: 1.037, 95% CI: 1.003–1.072, *P* = 0.034) had effects on prognosis. Risk score stood out as an independent prognostic factor in both the training set and the validation set.

### The Prognosis Analysis of Patients Received Different Treatments

196 OSCC patients received radiotherapy and 191 OSCC patients received chemotherapy. Supplementary Figure 4 showed the survival analysis of patients receiving different treatments. The results showed that there was no significant difference in survival between radiotherapy and pharmacotherapy. Correspondingly, Supplementary Figure 5 showed that there was no significant difference in the risk scores of patients undergoing radiotherapy and chemotherapy.

### Immune Infiltration Analysis

**FIGURE 5:**
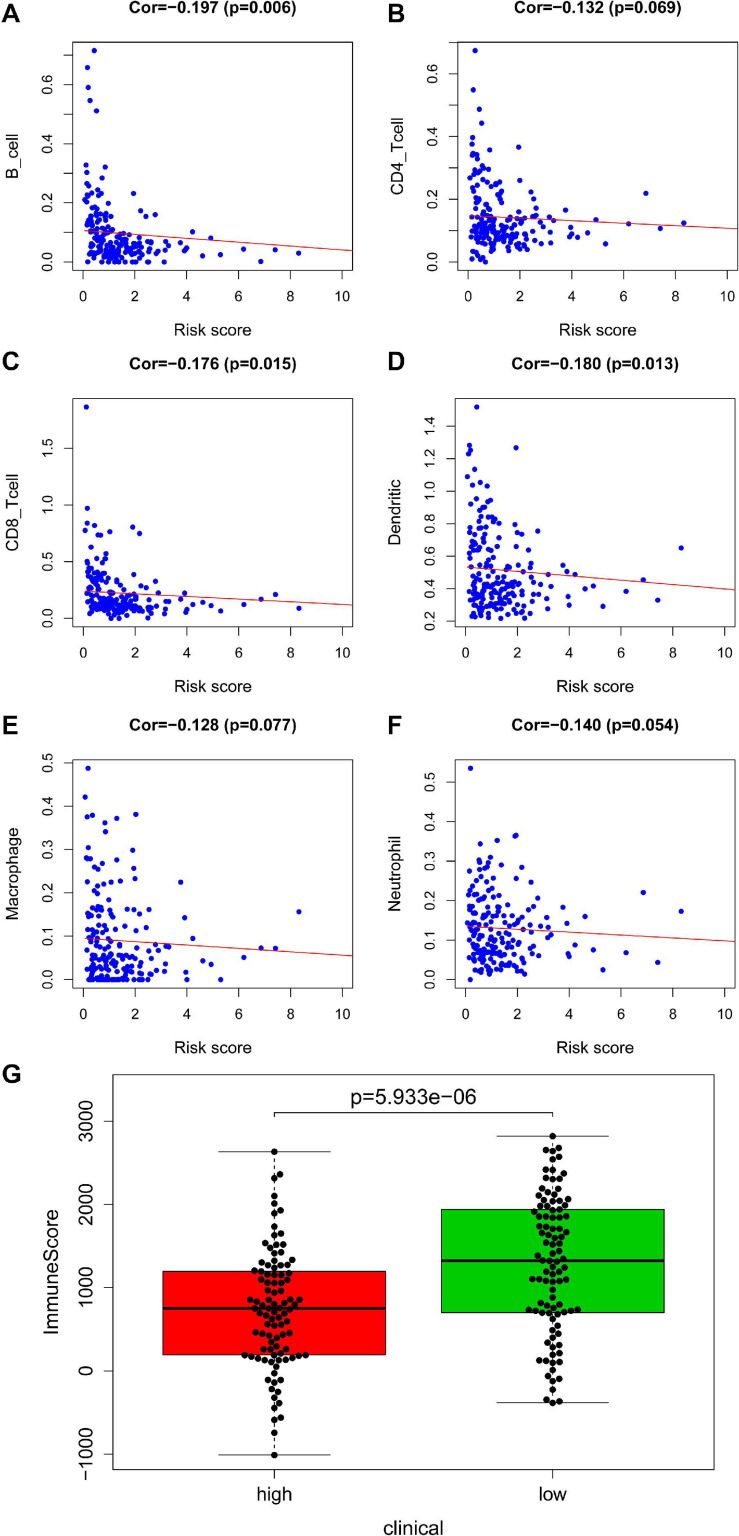
Analysis of immunity based on train set. Panels **(A–F)** indicates scatter diagram for risk score vs. tumor-infiltrating immune cells such as B cell, CD4+ T cell, CD8+ T cell, dendritic cell, macrophage and neutrophil, and corresponding linear regression lines. Panel **(G)** indicates box plot for immunescores between the high and low risk groups.

In training set, tumor-infiltrating immune cells including B cell (correlation (cor) = −0.197, *P* = 0.006), CD4+ T cell (cor = −0.132, *P* = 0.069), CD8+ T cell (cor = −0.176, *P* = 0.015), dendritic cell (cor = −0.180, *P* = 0.013), macrophage (cor = −0.128, *P* = 0.077), and neutrophil (cor = −0.140, *P* = 0.054) were negatively correlated with risk score (Figures 5A–F). This suggested that as the risk score increased, the patient’s immune capacity decreased. It was worth mentioning that there was a significant difference in immune scores between the high and low risk groups (*P* = 5.933e–06) (Figure 5G).

**FIGURE 6:**
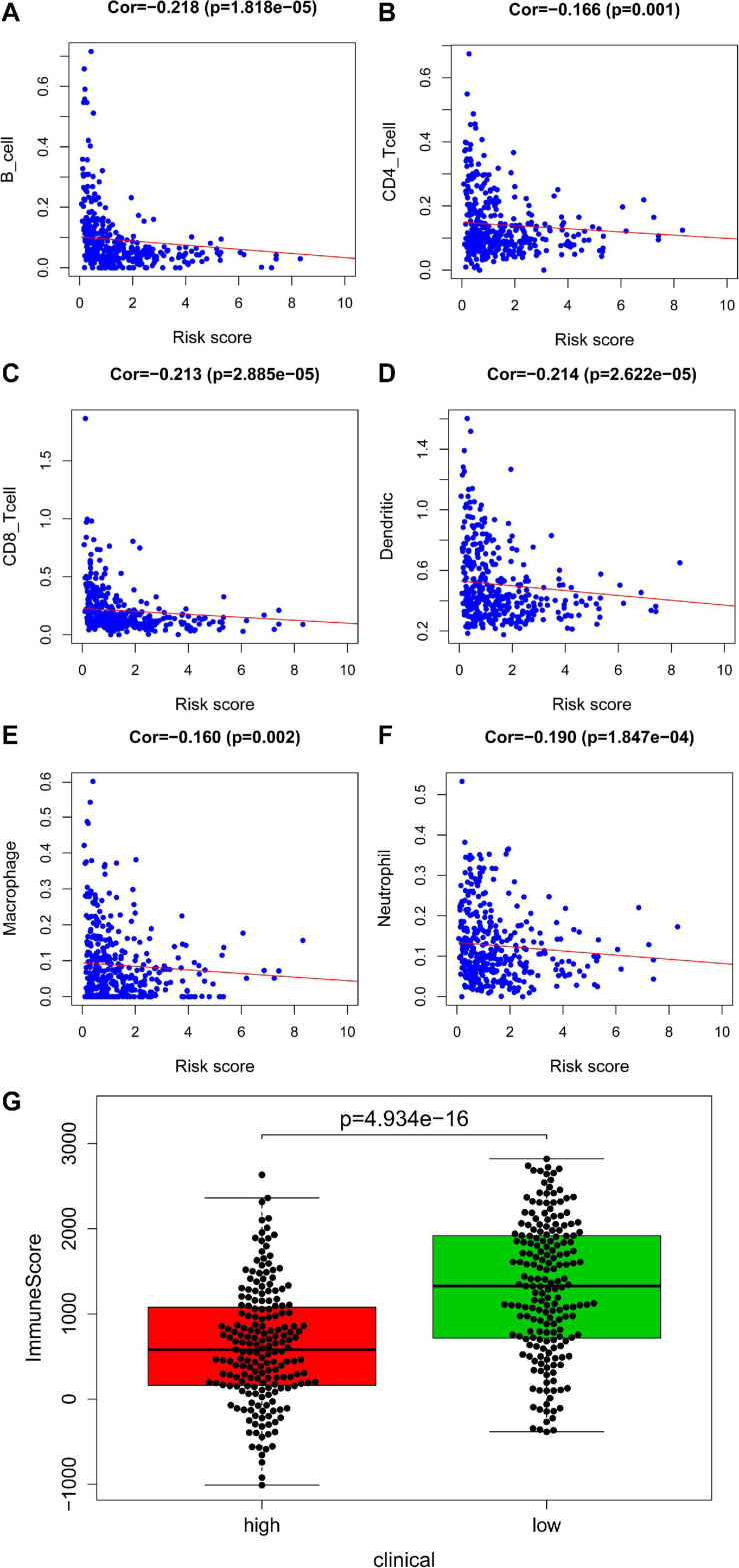
Analysis of immunity based in validation set. Panels **(A–F)** indicates scatter diagram for risk score vs. tumor-infiltrating immune cells such as B cell, CD4+ T cell, CD8+ T cell, dendritic cell, macrophage and neutrophil, and corresponding linear regression lines. Panel **(G)** indicates box plot for immunescores between the high and low risk groups.

An immune infiltration analysis based on the validation set was performed to confirm the correlation between immune cells and risk scores. The higher the patient’s risk score, the lower the infiltration of immune cells, including B cell (cor = −0.218, *P* = 1.818e–05), CD4+ T cell (cor = −0.166, *P* = 0.001), CD8+ T cell (cor = −0.213, *P* = 2.885e–05), dendritic cell (cor = −0.214, *P* = 2.622e–05), macrophage (cor = −0.160, *P* = 0.002), and neutrophil (cor = −0.190, *P* = 1.847e–04) infiltration, were negatively correlated with patient’s risk scores (Figures 6A–F). Figure 6G demonstrated that a significant difference between the two groups (*P* = 4.934e–16).

### Construction of the Nomogram, Internal Validation, GSEA, and Pathological Tissue

**FIGURE 7:**
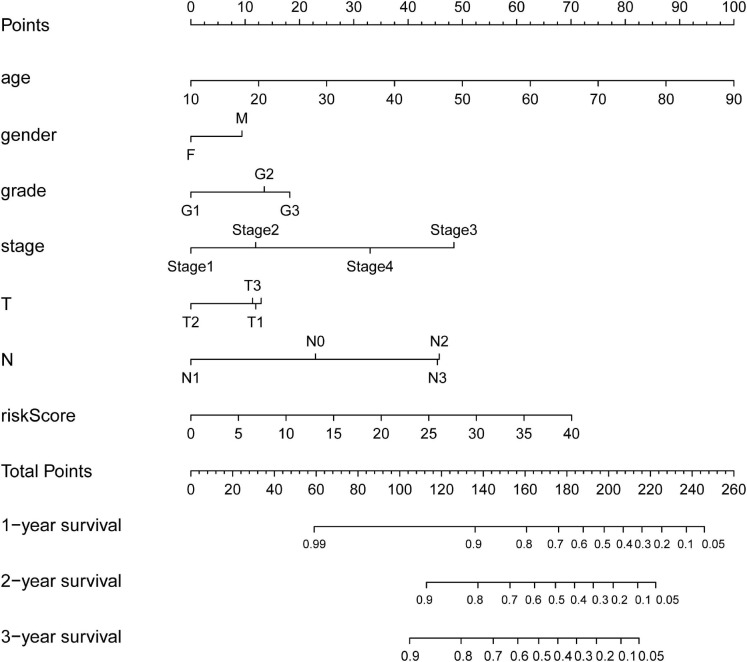
Nomograms for predicting OSCC Survival. The nomogram incorporating age, gender, grade, stage, T, N, and risk score for predicting the 1-, 2-, and 3-year OS of OSCC patients.

**FIGURE 8:**
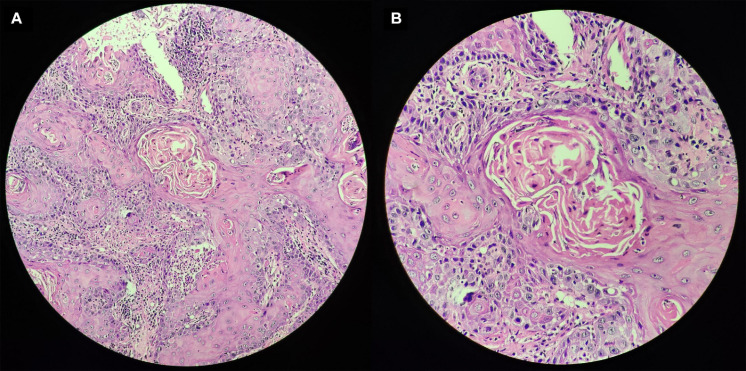
Pathological tissues of OSCC patients based on hematoxylin–eosin staining. Panel **(A)** indicates original magnification 200-fold, and panel **(B)** indicates original magnification 400-fold.

As shown in Figure 7, nomogram which included age, gender, stage, T, N and the risk score were constructed to predict the 1-, 2-and 3-year OS of patients with OSCC. The internally validated C-index were 0.7, indicating that the key genes combination performed well in clinical application. Calibration curve were in Supplementary Figure 6. Furthermore, the top 10 KEGG pathways with *P*-value < 0.05 obtained in GSEA such as alpha linolenic acid metabolism, amino, and nucleotide sugars metabolism, arachidonic acid metabolism, cysteine, and methionine metabolism were shown in Supplementary Figure 7 (training dataset). The top pathways, including alpha linolenic acid metabolism, arginine and proline metabolism, arachidonic acid metabolism and so on, with *P*-value < 0.05 based validation set were visualized in Supplementary Figure 8. The pathways obtained in the training set and the validation set overlap. In order to provide clear histological information, the pathological tissues of OSCC patients were selected in this study and case analysis graphs of 200-fold and 400-fold were performed under hematoxylin–eosin staining in Figure 8.

## Discussion

The study was conducted based on OSCC and normal oral samples from TCGA, as well as metabolic related genes in the context of KEGG pathways. 390 OSCC samples were divided into training set and validation set. Training set randomly obtained was used to construct the prognosis model, while validation set to test. The screened DEGs with | log_2_ FC| > 0.5 and FDR < 0.05 were subjected to regression analysis to construct prognostic models based on the train set. Specifically, 452 metabolism-related DEGs including 209 down-regulated genes and 243 up-regulated genes were conducted to regression analysis to construct prognostic models. Univariate COX regression analysis, Lasso regression analysis and multivariate regression analysis successively identified key gene, which was used to construct the prognostic model. Finally, nine key genes PIP5K1B, MINPP1, PYGL, AGPAT4, ENTPD1, NAGK, HADHB, CA12, and CA9 were obtained, which was the core content of this study. Multivariate COX regression analysis was used to calculate the risk score based on the nine genes, and the OSCC samples in the training set and the validation set were divided into two groups (“high-risk” vs. “low-risk”) based on the median risk score of the patients in the training set. Remarkably, gene signatures including PIP5K1B, MINPP1, AGPAT4, HADHB, and CA12 has been shown to be significantly associated with survival (^∗^*P* < 0.05 was considered statistically significant) based on the forest plot of nine key genes. Carbonic anhydrase 9 (CA9/CAIX) was a specific anoxic biomarker that was expressed in many cancers. Studies of OSCC had shown that CA9 was significantly up-regulated (Lin et al., 2018) and the CA9 mRNA level significantly increased (Eckert et al., 2019), and CA9 was considered to be an independent prognostic factor associated with poor prognosis (Peterle et al., 2018). CA9 was also expressed significantly in malignant oral disorders (mostly oral leukoplakia), which contributes to individualized prediction (Zhang et al., 2017). In a cohort study of head and neck squamous cell carcinoma, Based 9-gene model including CA9 was considered to be able to classify the disease for better clinical treatment (Clatot et al., 2014). In addition, CA9 may mediate the role of betel nut in OSCC genesis (Yang et al., 2014). The expression of CA9 was not only affected by hypoxia, but also regulated by proliferation-related signals (Klimowicz et al., 2013), which suggested us combined analysis may be more valuable. PIP5K1B (phosphatidylinositol-4-phosphate 5-kinase type 1 beta) was thought to be associated with focal turnover, neurite formation and oxidative stress response, and may be regulated by AMPK (AMP-activated protein kinase) and PKC (protein kinase C) (van den Bout et al., 2013). It was thought to be associated with lung adenocarcinoma (Xu et al., 2016) and Hodgkin’s lymphoma (Huang et al., 2018). MINPP1 and PTEN (phosphatase and tensin homolog) have overlapping functions: the ability to remove 3-phosphate from inositol phosphate substrates (Gimm et al., 2001). Hermans et al. (2004) showed in their study of prostate cancer xenografts and cell lines that PTEN inactivation may be accompanied by the loss of one MINPP1 allele. PTEN was a recognized cancer suppressor gene that mutates at high frequencies in many cancers. A study of 44 oral tongue squamous cell carcinoma formalin fixed paraffin embedding cases with high throughput mutation analysis showed that PTEN (14%) had missense, non-sense, and code shift mutations (Alsofyani et al., 2020). Many studies on the underlying mechanisms of OSCC are based on the down-regulation of PTEN (Liu et al., 2019; Zhang et al., 2020). However, the potential association between MINPP1 and OSCC needs to be supplemented. At present, we mainly understand that this gene may be related to the pathogenesis of malignant follicular thyroid tumors (Gimm et al., 2001). PYGL, which encodes a protein that can catalyze the cleavage of alpha-1,4-glucosidic bonds, was reported in colorectal cancer (Bien et al., 2019), thyroid cancer (Gomez-Rueda et al., 2016), and glioblastoma (Abbadi et al., 2014). AGPAT4, ENTPD1, HADHB, CA12, CA9 was reported in colorectal cancer. Overall, how were the key genes involved in cancer including OSCC in occurrence and progression to be supplemented.

The following works were mainly based on training set (*N* = 195), and validation set (*N* = 390). Kaplan–Meier Survival analysis and ROC curves were used for objective evaluation of the prognostic model. Kaplan–Meier survival curves estimating OS: Over time, the high-risk group was suggested to have a lower survival rate than the low-risk group. Independent prognostic analysis based on the training set indicated that the risk score (AUC = 0.809) was superior as an independent prognostic factor compared to other clinical characteristics including age, gender, grade, stage, T, and N. For validation set, the AUC of the clinical characteristics included in the study (age, gender, grade, stage, T, and N) was lower than the risk score (AUC = 0.722), indicating that the risk score had more accurate prediction of potential. Age, as an important independent prognostic factor, has always been concerned by researchers. Relevant research (Kheirandish et al., 2020) showed that the age of all enrolled samples (20–90 year old) was demonstrated to have strong effect on promoter methylation of studied genes. Hypomethylation of DKK2 and DKK4 genes in higher grades of OSCC samples may indicate the pivotal role of their expression in tumor cells invasion and progression through modulation of Wnt signaling pathway (Kheirandish et al., 2020). However, the clinical outcomes of meta-analysis demonstrated that young adults with OSCC experienced similar oncologic outcomes as older patients with OSCC after definitive treatment (Lee et al., 2020). Based on our study, it showed that the age appears to do better than risk score in the validation set, it indicating overfitting in the training set. This multiple contradictory conflict between molecular and clinical level is still worth further study. Kaplan-Meier survival curves of survival probability for the high-risk group decreased more significantly and the survival probability of the two groups was significantly different. Univariate and multivariate COX regression analyses were conducted using the “survival” package for exploring prognosis-related clinical factors on the prognostic model. The independent prognostic factors obtained by regression analysis were different based on the training and validation set. However, the risk score stood out as an independent prognostic factor in both the training set and the validation set, suggesting that the risk score was of interest.

To understand the relationship between the immune status of tumor microenvironment and risk score, Analysis of Tumor-infiltrating immune cells was performed. The interactions between tumor-infiltrating immune cells and tumors were complex, and the negative correlation between risk scores and tumor-infiltrating immune cells (B cells, CD4^+^ T cells, CD8^+^ T cells, neutrophils, macrophages, and dendritic cells) suggested an association between risk scores and infiltration of immune cells. Moreover, there was a significant difference in immune scores between the high and low risk groups. The higher risk score, the lower the infiltration of immune cells. What was noteworthy was that ENTPD1/CD39 (ectonucleoside triphosphate diphosphohydrolase 1) encodes a plasma membrane protein that hydrolyzes high-energy phosphate bonds, and some regulatory T cells with ENTPD1/CD39 overexpression played an important role in the breast cancer microenvironment (Gourdin et al., 2018). Simoni et al. (2018) studied CD8^+^ T cells in colorectal cancer and lung cancer and showed that ENTPD1/CD39 was deficient in bystander T cells. Alpha linolenic acid metabolism, arginine and proline metabolism, and arachidonic acid metabolism were is identified with a significant difference between the high-risk group and a low risk group. Based on GSEA, and these pathways were closely related to OSCC. The pathways identified in the training set and the validation set, for example, alpha linolenic acid metabolism. Finally, we included age, gender, grade, stage, T, N, and risk score to construct a nomogram to predict 1-, 2-, and 3-year survival for OSCC patients. The nomogram may contribute to a more accurate and individualized prediction for the prognosis of newly diagnosed OSCC patients, and provide advice for clinical treatment and follow-up observations. This study may provide new insights for further study of OSCC. However, there was no denying that there may be bias due to the small sample sizes, more studies are needed to validate our results.

In conclusion, a prognostic model of nine survival-associated metabolic gene signatures was identified and constructed using univariate COX regression analysis, Lasso regression analysis and multivariate regression analysis. The efficacy of the prognostic model was evaluated based on the training set and validation set. Both the Kaplan–Meier survival analysis and the ROC curve showed that the prognosis model performed well. Univariate and multivariate COX regression analysis based on the training set and validation set indicated that the risk score was superior as an independent prognostic factor compared to other clinical characteristics. Furthermore, tumor-infiltrating immune cells were negatively correlated with risk score, and there was a significant difference in immune scores between the high and low risk groups in training set and validation set. The differential pathways between the high and low risk groups were identified based on the results of GSEA. A nomogram incorporating some clinical characteristics and risk score was constructed to predict 1-, 2-, and 3-year survival rates for OSCC patients. The study may contribute to a more accurate and individualized prediction for the prognosis of newly diagnosed OSCC patients, and provide advice for clinical treatment and follow-up observations.

## Data Availability Statement

The raw data were downloaded from The Cancer Genome Atlas database (TCGA, https://portal.gdc.cancer.gov/).

## Ethics Statement

Pathological tissues of OSCC was consented by the patient, and approved by medical ethical committee of the Taihe Hospital, Hubei University of Medicine (No. 2021KS012).

## Author Contributions

Y-MN and CZ conceived and designed this study. Z-DH and T-YC carried out the analysis procedure. Z-DH, Y-YY, and T-YC analyzed the results. T-YC and Y-FZ contributed analysis tools. T-YC, Y-YY, and Z-DH participated in the manuscript writing. All the authors reviewed the manuscript.

## Conflict of Interest

The authors declare that the research was conducted in the absence of any commercial or financial relationships that could be construed as a potential conflict of interest.

## Footnotes

1 http://software.broadinstitute.org/gsea/downloads.jsp#msigdb

2 https://cistrome.shinyapps.io/timer/
