# Supplementary material for: Construction of Prognostic Risk Prediction Model of Oral Squamous Cell Carcinoma Based on Nine Survival-Associated Metabolic Genes
Source: Front Physiol. 2021 Mar 16;12:609770. doi: 10.3389/fphys.2021.609770 (PMC8011568; doi:10.3389/fphys.2021.609770)
Supplement: Supplementary file 1 [file Table_1.DOCX]

**Legend of Supplementary Figures**

**Supplementary Figure 1** A heatmap showing expression of DEGs in tumor and normal samples.

**Supplementary Figure 2** Volcano plot.

**Note:** The genes with |log_2_ fold change (FC)|>0.5 and P<0.05 were thought to be primary DEGs. The red node indicated that the gene is up-regulated, the green node indicated that the gene is down-regulated, and the black node indicated that the gene is excluded.

**Supplementary Figure 3** Feature selection using the Lasso regression model.

**Note:** A indicates a coefficient sectional view plotted against the log (λ) magnitude. B indicates process of variable selection in Lasso regression analysis based on 1,000 cross-validations. In the LASSO COX model, the minimum standard is adopted to obtain the value of the super parameter λ by 10-fold cross-validation.

**Supplementary Figure 4** Survival analysis of patients receiving radiotherapy and chemotherapy

**Supplementary Figure 5** Risk scores of patients undergoing radiotherapy and chemotherapy

**Supplementary Figure 6** Calibration curve.

**Supplementary Figure 7** Gene Set Enrichment Analysis based on training dataset.

**Supplementary Figure 8** Gene Set Enrichment Analysis base on validation dataset

**Supplementary Figure 1** A heatmap showing expression of DEGs in tumor and normal samples

**
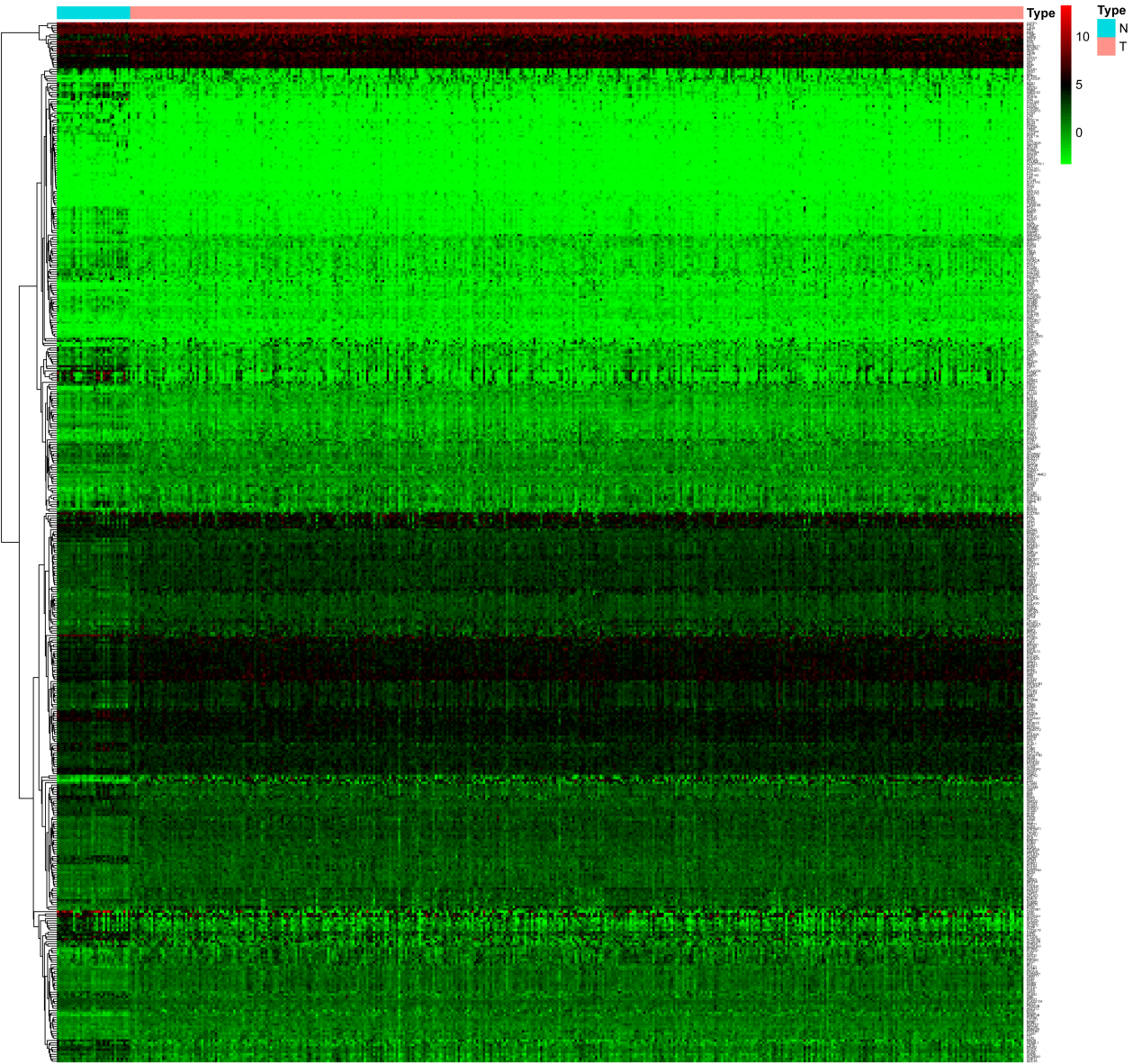
**

**Supplementary Figure 2** Volcano plot

**
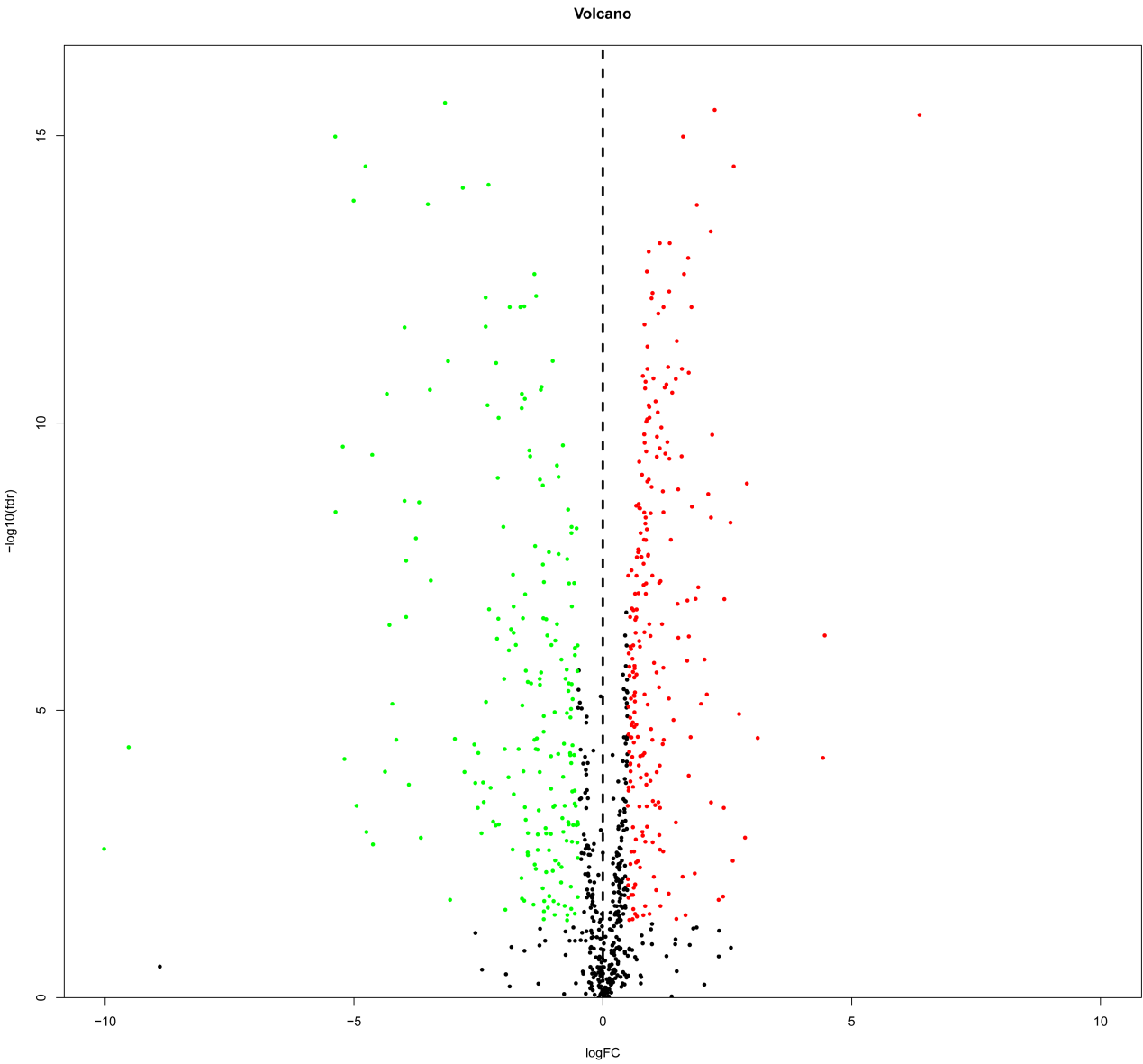
**

**Note:** The genes with |log_2_ fold change (FC)|>0.5 and P<0.05 were thought to be primary DEGs. The red node indicated that the gene is up-regulated, the green node indicated that the gene is down-regulated, and the black node indicated that the gene is excluded.

**Supplementary Figure 3** Feature selection using the Lasso regression model.

**
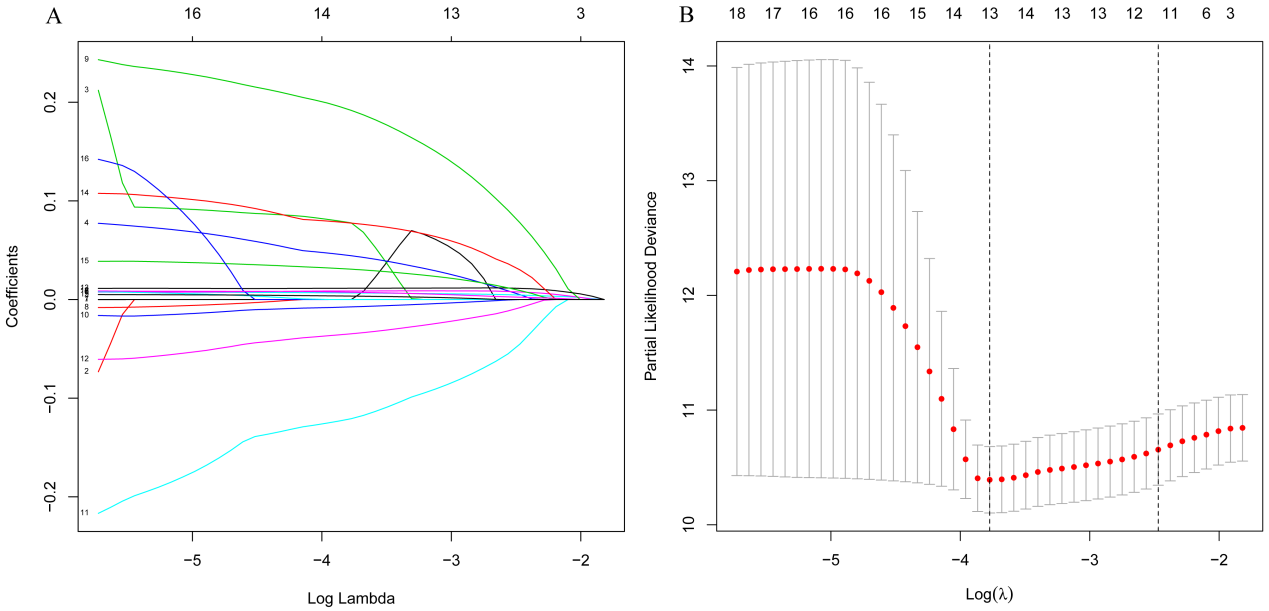
**

**Note:** A indicates a coefficient sectional view plotted against the log (λ) magnitude. The horizontal axis represents the log value of the independent variable lambda, and the vertical axis represents the coefficient of the independent variable. B indicates process of variable selection in Lasso regression analysis based on 1,000 cross-validations. In the LASSO COX model, the minimum standard is adopted to obtain the value of the super parameter λ by 10-fold cross-validation.

**Supplementary Figure 4** Survival analysis of patients receiving radiotherapy and chemotherapy

**
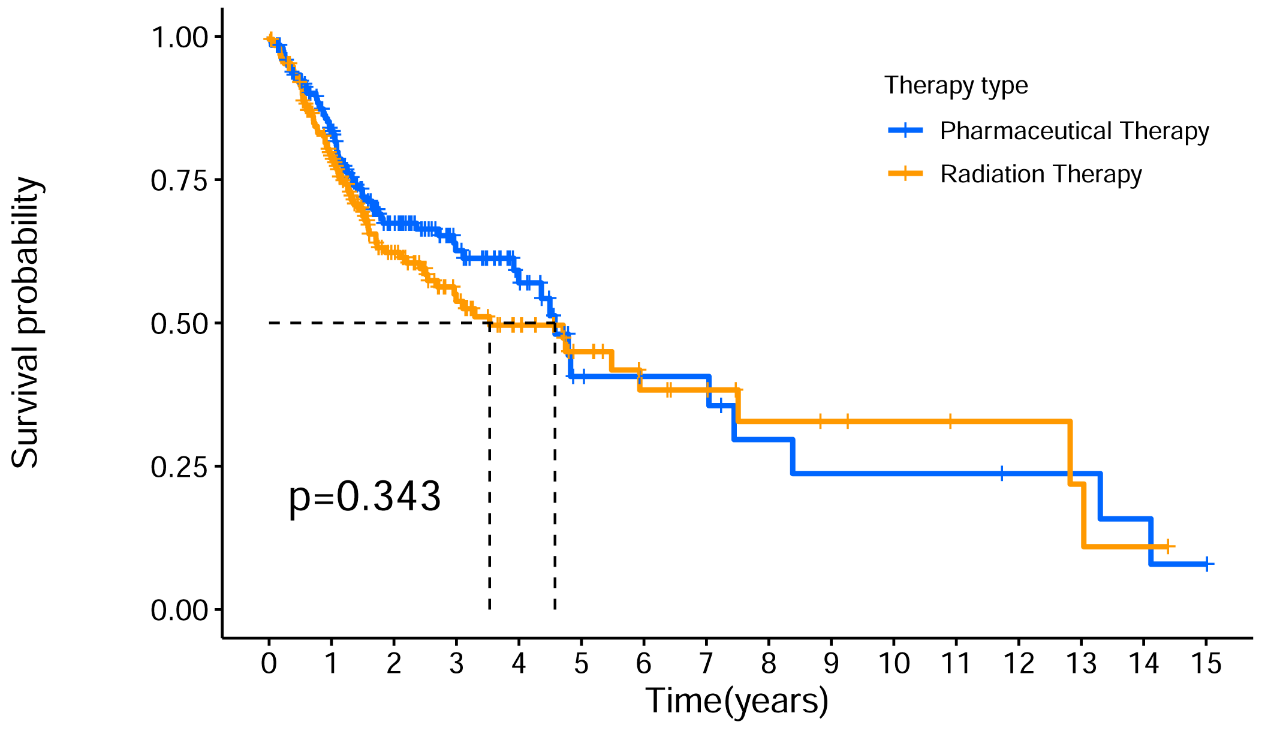
**

**Supplementary Figure 5** Risk scores of patients undergoing radiotherapy and chemotherapy

**
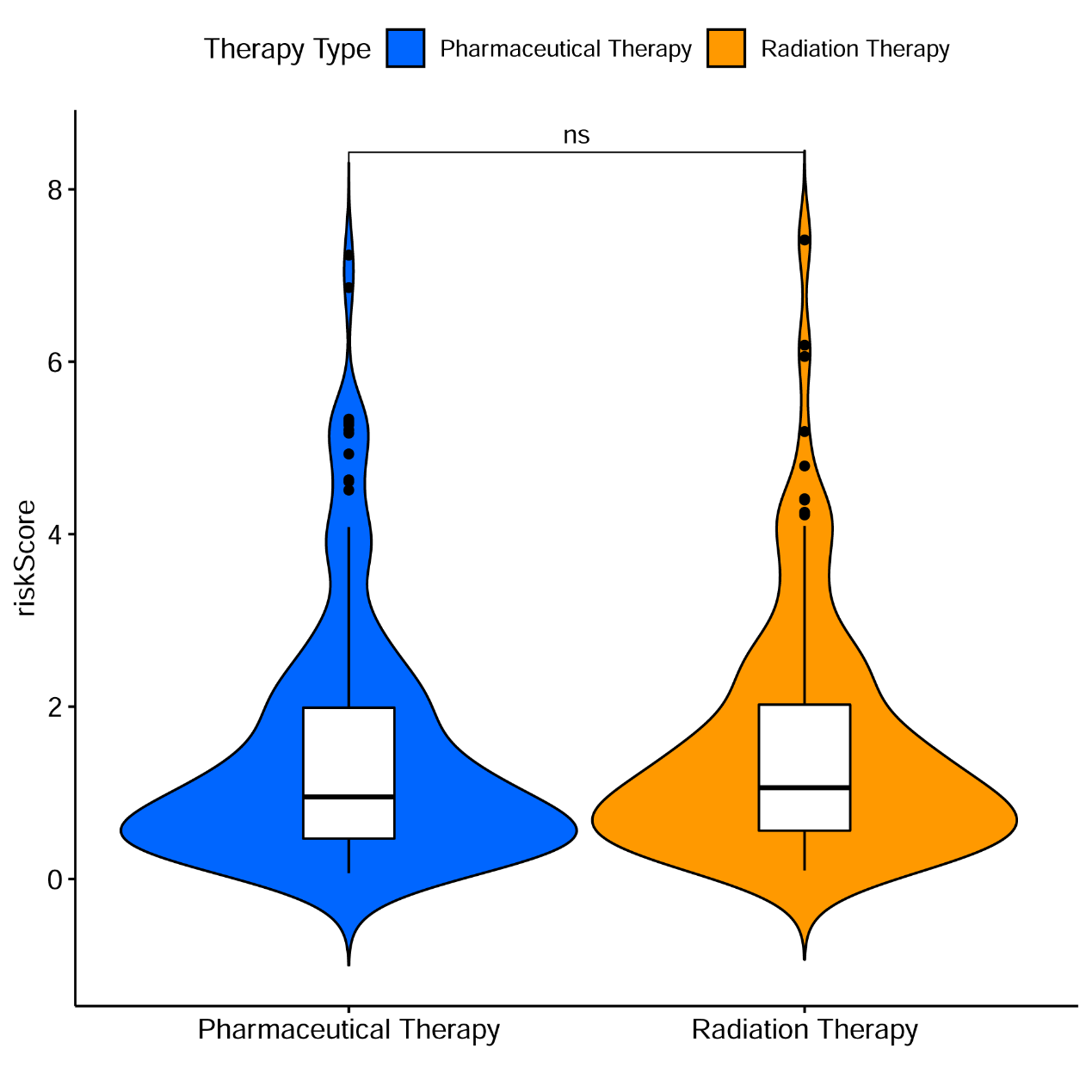
**

**Supplementary Figure 6** Calibration curve

**
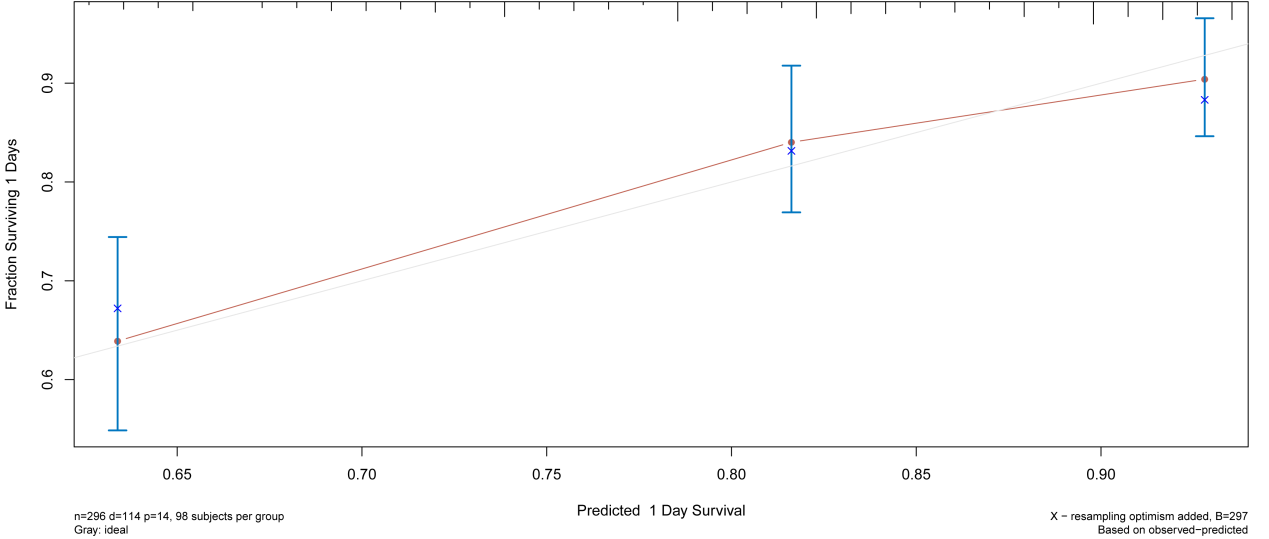
**

**Supplementary Figure 7** Gene Set Enrichment Analysis based on training dataset

**
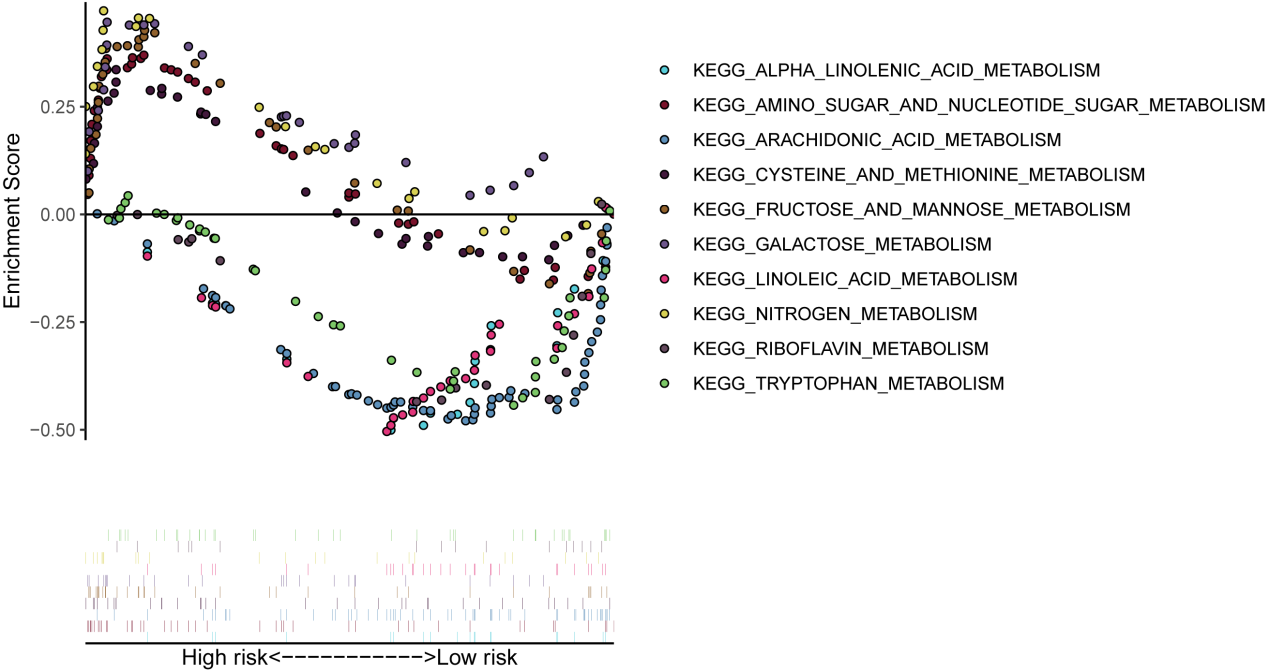
**

**Supplementary Figure 8** Gene Set Enrichment Analysis base on validation dataset

**
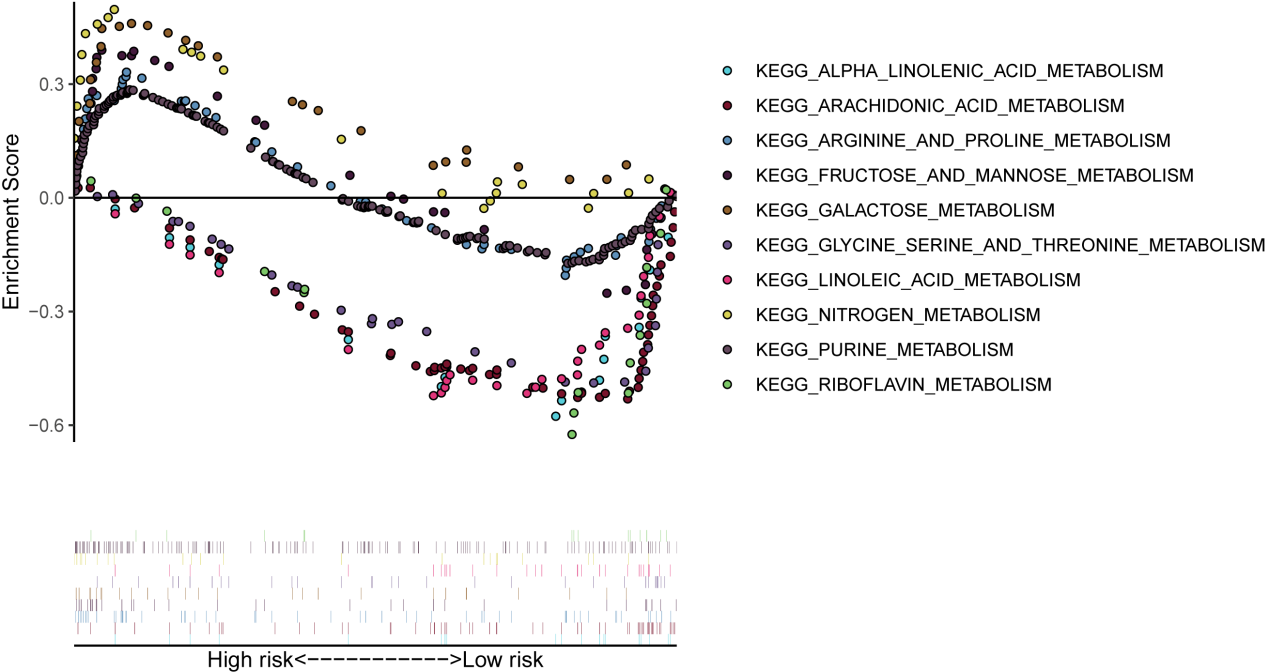
**
